# Signatures of Selection for Resistance/Tolerance to *Perkinsus olseni* in Grooved Carpet Shell Clam (*Ruditapes decussatus*) Using a Population Genomics Approach

**DOI:** 10.1111/eva.70106

**Published:** 2025-05-13

**Authors:** Inés M. Sambade, João Estêvão, Marina Pampín, Andreia Cruz, Eric Guévélou, Andrés Blanco, Francisco Câmara, Jessica Gómez‐Garrido, Fernando Cruz, Luca Bargelloni, Stefano Carboni, Tyler Alioto, Benjamin Costas, Sergio Fernández‐Boo, Paulino Martínez

**Affiliations:** ^1^ Department of Zoology, Genetics and Physical Anthropology, Facultad de Veterinaria, Campus Terra Universidade de Santiago de Compostela Lugo Spain; ^2^ Aquatic Animal Health (A2S) CIIMAR—University of Porto Porto Portugal; ^3^ Oceano Fresco S.A Nazaré Portugal; ^4^ Centre Nacional d'Anàlisi Genòmica (CNAG) Barcelona Spain; ^5^ Universitat de Barcelona (UB) Barcelona Spain; ^6^ University of Padova Padova Italy; ^7^ IMC Foundation Sardinia Italy

**Keywords:** 2bRAD‐seq, genetic structure, genome assembly, perkinsosis, population genomics, resistance/tolerance

## Abstract

The grooved carpet shell clam (*Ruditapes decussatus*) is a bivalve of high commercial value distributed throughout the European coast. Its production has suffered a decline caused by different factors, especially by the parasite *Perkinsus olsenii*. Improving production of *R*. *decussatus* requires genomic resources to ascertain the genetic factors underlying resistance/tolerance to *P. olsenii*. In this study, the first reference genome of *R*. *decussatus* was assembled through long‐ and short‐read sequencing (1677 contigs; 1.386 Mb) and further scaffolded at chromosome level with Hi‐C (19 superscaffolds; 95.4% of assembly). Repetitive elements were identified (32%) and masked for annotation of 38,276 coding‐ and 13,056 non‐coding genes. This genome was used as a reference to develop a 2bRAD‐Seq 13,438 SNP panel for a genomic screening on six shellfish beds distributed across the Atlantic Ocean and Mediterranean Sea. Beds were selected by perkinsosis prevalence and the infection level was individually evaluated in all the samples. Genetic diversity was significantly higher in the Mediterranean than in the Atlantic region. The main genetic breakage was detected between those regions (F_ST_ = 0.224), being the Mediterranean more heterogeneous than the Atlantic. Several loci under divergent selection (394 outliers; 261 genomic windows) were detected across shellfish beds. Samples were also inspected to detect signals of selection for resistance/tolerance to *P. olsenii* by using infection‐level and population‐genomics approaches, and 90 common divergent outliers for resistance/tolerance to perkinsosis were identified and used for gene mining. Candidate genes and markers identified provide invaluable information for controlling perkinsosis and for improving production of the grooved carpet shell clam.

## 1 Introduction

The grooved carpet shell clam (*Ruditapes decussatus*) is a bivalve species distributed throughout the coastal and estuarine areas of the Northeast (NE) Atlantic Ocean and the Mediterranean Sea (Juanes et al. 2012; Cordero et al. 2014). This species is of high economic and social importance, especially in southern Europe, with Portugal standing as the largest producer (FAO 2024). However, its production has suffered a decline caused by massive mortalities, attributed to a combination of different biotic (pathogens) and abiotic (heat waves and salinity stress) factors, poor management protocols, and the intensive culture of the non‐indigenous species *Ruditapes philippinarum*, which is prone to compete with endemic clams (Azevedo 1989; Ruano et al. 2015). The parasite *P. olsenii* was first reported in Europe in *R. decussatus* in 1989 after massive mortalities in Southern Portugal (Azevedo 1989), and since then, it has become a major issue for clam culture (Villalba et al. 2004). *P. olsenii* life cycle is based on trophozoites detected on the affected tissues of the host, as spherical cells with a large vacuole and a peripheral nucleus. These cells encompass vegetative proliferation by successive bipartitioning that, after cell wall rupture, release daughter cells. The daughter cells later enlarge, giving rise to mature trophozoites, which can infect new hosts after release through pseudo‐faeces (Bushek et al. 2002) or after tissue decomposition from the dead host. In this case, trophozoites develop a resistant state called hypnospore by enlarging and forming a new cell wall. After release into the seawater and under optimal conditions, they develop into pre‐zoosporangia, which can produce thousands of free and motile invasive biflagellated zoospores (Azevedo et al. 1990; Auzoux‐Bordenave et al. 1996; Villalba et al. 2004). All stages of the parasite life cycle are infective. Currently, collectors and farmers of this clam species face significant challenges of seed supply and recruitment from shellfish beds, together with important annual mortalities of adults close to commercialization caused by high prevalence of *P. olsenii* (Villalba et al. 2004; da Costa et al. 2020).

To counteract mortalities due to environmental factors and pathogenic infections, such as perkinsosis, breeding programs aimed at obtaining improved bivalve strains resilient to biotic and abiotic stress conditions have been launched in different bivalve species, such as oysters, mussels, and clams (Hollenbeck and Johnston 2018; Potts et al. 2021). Genetic diversity is a cornerstone for broodstock foundation to ensure long‐term genetic response and appropriate handling of genotype by the specific environment interactions in the areas of production (Gjedrem and Baranski 2009). A sufficiently large and genetically diverse broodstock also diminishes the risk of inbreeding depression and unintended selection (Duncan et al. 2013). Therefore, understanding genetic structure and population dynamics of the species throughout its distribution is essential for a sustainable production and for efficient breeding programs. Bivalves usually have a bipartite life cycle characterized by a dispersive, planktonic larval phase and a sedentary adult phase (D'Aloia et al. 2015). While larvae dispersal by marine currents during the planktonic phase contributes to homogenize populations (Hellberg 2009; Vera et al. 2022), oceanic barriers and fronts determine some differentiation across the whole genome (Hellberg 2009; Vera et al. 2022, 2023). Additional differentiation at specific genomic regions might be related to adaptation to diverse environmental factors across the species distribution range (Miller et al. 2019; Wu et al. 2020).

Population genetics studies of *R. decussatus* have been performed to date with a limited number of markers, and low, but significant, population differentiation has been detected throughout the distribution range, especially pronounced between Atlantic and Mediterranean regions (Cordero et al. 2014; Arias‐Pérez et al. 2016; Cruz et al. 2020). Three main genetic groups, corresponding to Atlantic, Western Mediterranean, and Eastern Mediterranean, were identified, with a main discontinuity separating the Atlantic and Mediterranean groups. However, mtDNA haplotypes placed Atlantic and Western Mediterranean groups closer regarding the Eastern Mediterranean. The most comprehensive study detected genetic differences between infected and non‐infected clams at several microsatellite loci, suggesting that resistance to the *P. olsenii* parasite could have a genetic basis (Cruz et al. 2020).

Despite the relevant information achieved, a whole genomic screening would provide more refined data regarding the factors shaping the genome of the species across its distribution. This is essential for disclosing the mechanisms of adaptation, either at a local or broader scale. Next generation sequencing (NGS) technologies have made affordable contiguous and consistent genome assemblies, which have been used to call and genotype thousands of single nucleotide polymorphisms (SNP) across the whole genome (Yue and Wang 2017; Houston et al. 2020; Yáñez et al. 2023). Restriction site associated DNA sequencing (RAD‐Seq) methodologies represent a powerful tool for genomic screening to be applied in breeding programs or population genomics studies (Robledo et al. 2018). RAD‐Seq technologies have been employed for population genomics studies in mollusks (Gutierrez et al. 2017; Yue et al. 2020), either to disclose the environmental factors shaping the genome (Vera et al. 2022, 2023) or to identify genomic regions and genetic markers associated with resilience to parasites (Vera et al. 2019; Hornick and Plough 2022; Sambade et al. 2022; Pampín et al. 2023).

In this study, the first chromosome‐level genome of *R*. *decussatus* was assembled and annotated, and it was further used for a preliminary evaluation of the genetic structure of the species using 2bRAD‐Seq. The study also focused on the genetic variation associated with resistance/tolerance to perkinsosis through individual evaluation of parasite load on shellfish beds affected by a wide range of perkinsosis incidence. A total of 90 markers and several candidate genes associated with perkinsosis resistance/tolerance were identified, representing invaluable information for controlling this parasitosis.

## 2 Materials and Methods

### 2.1 Whole Genome Sequencing (WGS)

#### 2.1.1 Long‐Read WGS

High molecular weight DNA was obtained with the E.Z.N.A. Mollusc DNA kit (Omega Biotek, USA) from one *R*. *decussatus* muscle foot from Algarve (Portugal) following the manufacturer's instructions. The sequencing libraries were prepared using the Ligation sequencing kit SQK‐LSK109 from Oxford Nanopore Technologies (ONT) and the quality parameters monitored by the MinKNOW platform version 4.1.2 in real time and base‐called with Guppy version 4.2.3 (Data S1).

#### 2.1.2 Short‐Read Whole Genome Sequencing

The short‐insert paired‐end libraries for the whole genome sequencing (WGS) were prepared using DNA from the same individual used for long‐read sequencing with a PCR‐free protocol using the KAPA HyperPrep kit (Roche) with some modifications (Data S1) and the quality was evaluated on an Agilent 2100 Bioanalyzer with the DNA 7500 assay (Agilent) for size and quantified by the Kapa Library Quantification Kit for Illumina platforms (Roche).

#### 2.1.3 Hi‐C Sequencing

Five *R. decussatus* males and five *R. decussatus* females were dissected, and several organs (mantle, haemocytes, gills, muscle, and gonads) were snap frozen in liquid nitrogen, pooled together, and maintained at −80°C until use. The different samples were pulverized using a mortar and pestle immersed in a liquid nitrogen bath. Hi‐C libraries were prepared using the Omni‐C kit (Dovetail Genomics), following the manufacturer's protocol (Data S1). The library was sequenced on NovaSeq 6000 (Illumina, 2 × 151 bp) following the manufacturer's protocol for dual indexing.

### 2.2 Genome Assembly

Before assembly, long‐ and short‐reads were preprocessed and filtered following a specific pipeline to ensure a minimum length and quality (Data S1 and Figure S1). The Omni‐C reads were mapped to the assembly using BWA‐MEM and pre‐processed using the Dovetail pipeline (https://omni‐c.readthedocs.io/en/latest/fastq_to_bam.html). After removal of PCR duplicates, they were scaffolded with YaHS30 v1.1 using default parameters, and the assembly error was corrected by 10 rounds of scaffolding. The resulting assembly (fRudec1) was evaluated with BUSCO v 5.4.0 (Simão et al. 2015) using the metazoan_odb10 lineage dataset and Merqury v 1.1 (Rhie et al. 2020) for consensus quality (QV) and k‐mer completeness. Finally, to compute the contiguity, CNAG's in‐house script Nseries.pl was used (https://github.com/cnag‐aat/assembly_pipeline/blob/v2.0.0/scripts/Nseries.pl).

### 2.3 Genome Annotation

#### 2.3.1 RNA‐Seq

For genome annotation, RNA‐Seq was carried out on gill, mantle, foot, haemocytes, and digestive gland using pools of 10 individuals for each tissue. Total RNA extraction was performed using the RNeasy mini kit (Qiagen) with DNase treatment. RNA quantity and quality were evaluated with the Qubit RNA BR Assay kit (Thermo Fisher Scientific) and the RNA integrity was estimated by using the RNA 6000 Nano Bioanalyser 2100 Assay (Agilent). Next, equimolar RNA pools of 10 individuals were used for library construction of each tissue after the evaluation of individual RNA extractions.

The RNA‐Seq libraries were prepared with the KAPA Stranded mRNA‐Seq Illumina Platforms Kit (Roche) following the manufacturer's recommendations (Data S1). The final library was validated on an Agilent 2100 Bioanalyser with the DNA 7500 assay.

#### 2.3.2 Repetitive Elements

Repeats present in the *R*. *decussatus* genome assembly were annotated with RepeatMasker v4‐1‐5‐0 (http://www.repeatmasker.org) using the custom repeat library available for Mollusca, after excluding those repeats that were part of repetitive protein families (performing a BLAST search against UniProt) (Data S1). Bedtools v2.31.1 (Quinlan and Hall 2010) was used to produce the final repeat‐masked version of the genome.

#### 2.3.3 Gene Annotation

Gene annotation was done by combining transcript alignments, protein alignments, and ab initio gene predictions following the CNAG structural genome annotation pipeline (https://github.com/cnag‐aat/Annotation_AAT) (Figure S2 and Data S1). RNA‐Seq reads obtained from several tissues, either sequenced specifically in this study (gill, mantle, foot, haemocytes, and digestive gland) or from public databases, were aligned against the genome with STAR v‐2.7.10a (Dobin et al. 2013) and used to generate transcript models with StringTie v2.2.1 (Niknafs et al. 2017). The TransDecoder program, which is part of the PASA package, was run on the PASA assemblies to detect coding regions in the transcripts. Additionally, the complete proteomes of *Crassostrea virginica* , *C. gigas* , *Mytilus coruscus*, *M. galloprovincialis* , and *M. edulis* were downloaded from Uniprot in April 2024 and aligned to the genome using Miniprot v0.6 (Li 2023). Ab initio gene predictions were performed on the repeat‐masked *R*. *decussatus* assembly with several programs with and without incorporating evidence from the RNA‐Seq data. Finally, all the data were combined into consensus CDS models using EvidenceModeler‐2.1 (EVM, Haas et al. 2008) and functional annotation was performed on the annotated proteins with Blast2go (Conesa et al. 2005). Additionally, UTRs and alternative splicing forms were annotated via two rounds of PASA annotation updates.

The annotation of non‐coding RNAs (ncRNAs) was obtained using the repeat‐masked version of the genome assembly and after removing RNA gene families identified with Infernal (Nawrocki and Eddy 2013) and tRNAscan‐SE (Chan and Lowe 2019) packages. Long non‐coding RNAs (lncRNAs) were identified after filtering protein‐coding genes to retain read clusters longer than 200 bp and not covered by more than 80% by a small ncRNA. Due to the lack of conservation between species, no functional annotation of the lncRNAs was performed. The final non‐coding annotation contains the lncRNAs and the sncRNAs.

### 2.4 Population Genomics Analysis

#### 2.4.1 Sampling and Parasite Load

**FIGURE 1:**
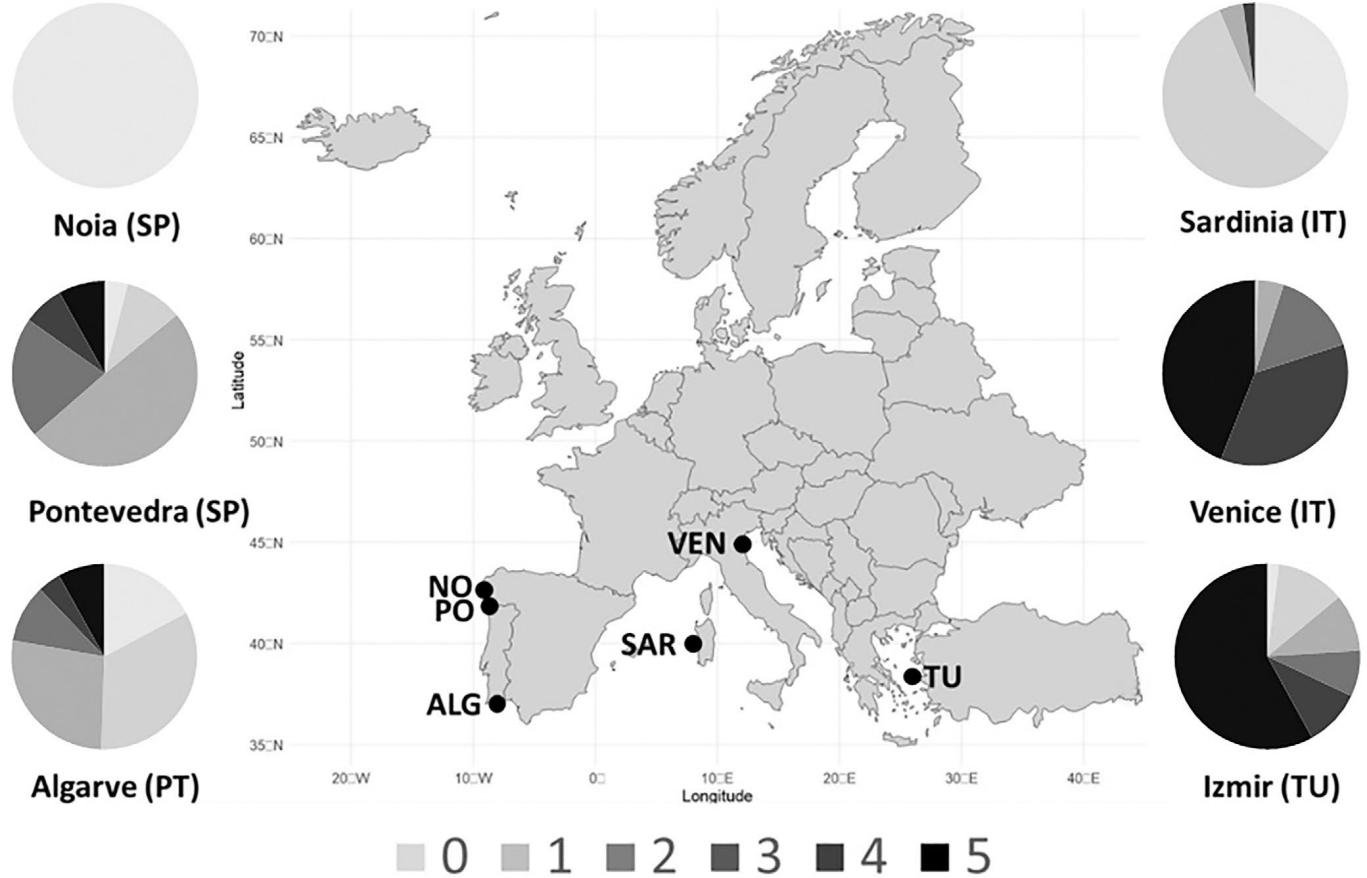
Geographical location of *R*. *decussatus* shellfish beds showing *P. olsenii* prevalence. The levels of infection are represented as pie charts, using a progressive grey scale based on RFTM categories. Prevalence of Venice was calculated as the averages of infected clams for each level from both samplings, 2019 and 2022.

Between 2019 and 2022, 100 clams per bed were collected and analyzed from six different shellfish beds with different perkinsosis prevalence: (i) three sampling sites from the NE Atlantic Ocean from Algarve (Portugal), Pontevedra (Spain), and Noia (Spain) (FAO Major Fishing Area 27s ‐ ATLANTIC, NORTHEAST; Subarea 27.9); and (ii) three from the Mediterranean Sea from Sardinia (Italy), Izmir (Türkiye), and two from Venice (Italy), which is the only site sampled repeatedly over time (FAO Major fishing Area 37, Mediterranean and Black sea; subareas 37.1.3, 37.3.1 and 37.2.1) (Table 1 and Figure 1). The two samples from the Venice lagoon (2019 and 2022) were collected to confirm the results observed in 2019, suggesting an admixture between Atlantic and Mediterranean regions (see Results).

**TABLE 1:** Main features of *R*. *decussatus* samples.

Sample code	Location	Country	N	Date	Prev. (%)	Perkins. status	Genotyped individuals from each infection level						% of individuals at each infection level						Coordinates
							L0	L1	L2	L3	L4	L5	L0	L1	L2	L3	L4	L5	
ALG	Algarve	Portugal	31	2020	83	LTA	6	5	5	5	3	7	17.2	33.3	27.3	10.1	4	8.1	37°01′11.9″N 7°50′11.3″W
NO	Noia	Spain	31	2020	0	Naïve	31	0	0	0	0	0	100	0	0	0	0	0	42°47′31.7″N 8°54′56.6″W
PO	Pontevedra	Spain	31	2020	96	LTA	4	5	5	9	4	4	4.0	10.1	49.5	21.2	7.1	8.1	42°25′41.0″N 8°41′15.3″W
SAR	Sardinia	Italy	30	2022	65	LTA	14	13	2	0	1	0	35.4	58.3	4.2	0	2.1	0	39°50′04.0″N 8°29′02.2″E
TU	Izmir	Turkey	30	2019	98	LTA	1	6	5	4	5	9	2	12	10	8	10	58	38°27′09.6″N 26°59′09.3″E
VEN19	Venice	Italy	30	2019	100	LTA	0	0	0	8	11	11	0	0	0	7	24	69	45°13′02.6″N 12°13′50.9″E
VEN22	Venice	Italy	32	2022	100	LTA	0	2	10	8	7	3	0	1	9	23	48	19	45°13′02.6″N 12°13′50.9″E

Abbreviations: Date, Year of sample collection; N, Number of genotyped individuals per population; Perkinsosis status, LTA (Long Term Affected) and Naïve (Non‐affected); Prev, Prevalence of infection.

Both hemigills from each clam were collected for *P. olsenii* load evaluation through histology and quantitative PCR (qPCR). Briefly, one hemigill was used for Ray's fluid thioglycollate medium (RFTM) diagnosis according to Ray (1966) using the following infection scale: level: 0—absence of parasite, 1—very slight infection, 2—slight infection, 3—moderate infection, 4—heavy infection, 5—critical infection. The other hemigill was placed in ethanol at 70% and stored directly at −20°C for DNA extraction used for qPCR parasite load evaluation according to García et al. (2022) with the *P. olsenii* specific primers designed by Ríos et al. (2020). After diagnosis, a total of 213 carpet‐shell clams were selected for genotyping trying to include all infection levels from each population (Table 1).

#### 2.4.2 RAD‐Seq SNP Genotyping

Total DNA was extracted from foot muscle (for genotyping) or gill (for qPCR) using the E.Z.N.A. Mollusc DNA kit (Omega Biotek, USA) following manufacturer instructions. SNP identification and selection, as well as genotyping and validation protocols followed Maroso et al. (2019). Briefly, AlfI IIb restriction enzyme (RE) was used to construct the 2b‐RAD libraries following the protocol by Manuzzi et al. (2019); samples were evenly pooled for sequencing in Illumina Next‐Seq500 including 90 individuals per run at the Genomics Platform of Universidad de Valencia (Spain). Then, home‐made scripts were used to cut and remove fastq sequences of unexpected length and to filter out sequences that did not include the Alf1 restriction site in the right position. Individuals with < 250,000 reads were discarded. The assembled *R*. *decussatus* genome was used as reference to align reads from each individual using Bowtie 1.1.2 (Langmead et al. 2009), allowing a maximum of three mismatches and a unique genome alignment (−v 3 −m 1). STACKS 2.0 (Catchen et al. 2013) was then used to call SNPs and genotype a common set of markers in the whole dataset, applying the marukilow model with default parameters in the gstacks module. This SNP panel was further filtered using STACKS 2.0 and PLINK 1.9 (Purcell et al. 2007) by applying the following criteria: (i) minimum allele count (MAC) ≥ 3 in the whole sample; (ii) depth > 6 reads (iii) genotyped in > 60% individuals in the whole sample; (iv) conformance to Hardy–Weinberg equilibrium (HWE; *p* > 0.05) in at least three shellfish beds across the whole collection; and (v) selection of the most polymorphic SNP within each RAD‐tag. Bioinformatic support for these analyses was provided by CESGA Supercomputer Center of Galicia.

#### 2.4.3 Genetic Markers and Genomic Regions Under Divergent Selection

We applied a method based on haplotype differentiation between population pairs around focal SNPs to detect signals of divergent selection for the two scenarios tested, geographic origin and perkinsosis infection status. Extended haplotype homozygosity (EHH) is a method used to detect selection signatures by measuring the persistence of haplotype homozygosity around a focal variant. Under directional selection, beneficial alleles rapidly increase in frequency, carrying linked haplotypes with them, which results in an extended region of high homozygosity (Gautier et al. 2017). Selection signals can be further investigated using the cross‐population EHH (xp‐EHH) test, which compares the decay of haplotype homozygosity between two populations to identify loci under selection in one population relative to the other. Alternatively, the Rsb test provides a standardized measure of selection by comparing the integrated EHH scores (iES) between populations.

In our study, we applied the Rsb test, as it is well suited for identifying selection signatures when the genetic structure between populations is not high. Haplotype phasing was performed separately for each chromosome and sample using SHAPEIT5 (Hofmeister et al. 2023) from VCF files, rendering haplotypes of variable size depending on chromosomes. The Rsb test was then applied to all population pairs, and significant genomic windows were identified when the standardized score exceeded 2.33 (*p* < 0.01). Within each significant window, the SNP with the highest signal (focal SNP) was selected for further analysis of genetic diversity and population structure using outlier loci associated with divergent selection.

For the geographic scenario, a total of 15 population pairwise comparisons were performed within the Atlantic Ocean (NO, PO, ALG; 3 comparisons), within the Mediterranean Sea (SAR, VEN, TUR, 3 comparisons), and between Atlantic and Mediterranean regions (9 comparisons). All SNPs for divergent selection detected in the three scenarios were pooled (eliminating duplications) to obtain the total set of SNPs showing significant divergence between any population pair in the total sampling.

For the perkinsosis infection scenario, we compared samples across infection levels using populations with a similar infection profile and from the same geographic area, to avoid the geographic differentiation component that could bias the results. To gain statistical power (sample size), we considered three main infection levels: low (L0, L1), moderate (L2, L3) and heavy (L4, L5). This approach assumes a similar selective pressure on the individuals from the populations compared, which could be expected considering their age (adults) and the previous records of perkinsosis in those populations. Accordingly, for the infection‐level approach, we used the information of PO and ALG from the Atlantic Ocean and of VEN and TU from the Mediterranean Sea. Common outliers and overlapping genomic windows across the two comparisons were identified and used to detect the most consistent candidate genes through gene mining.

#### 2.4.4 Genetic Diversity

Genetic diversity per sample was estimated using expected (He) and observed (Ho) heterozygosity, and allelic richness (Ar), computed using the rarefaction method to correct the bias due to sample size. These analyses were performed with the DiveRsity R package v. 1.9 using the ‘basicStats’ function (Keenan et al. 2013). Conformance to HWE was evaluated with an exact test implemented in the R package Genepop 4.7.5 (Rousset 2008). The sense and magnitude of the deviation from random mating was estimated with F_IS_ (intrapopulation fixation index) with exact tests using Genepop 4.7.5.

#### 2.4.5 Genetic Structure

Global and pairwise population differentiation (F_ST_; Weir and Cockerham 1984) were estimated using different grouping criteria. Pairwise F_ST_ between populations, as well as global F_ST_ for the whole dataset and for each region and their significance were obtained with the ‘Fst’ function of the R package Genepop 4.7.5 (Raymond and Rousset 1995).

StructureSelector software (Li and Liu 2018) was used to obtain K estimators and CLUMPAK outputs (Kopelman et al. 2015). Three K estimators were used to identify the most likely number of clusters: the deltaK *ad hoc* estimator (Evanno et al. 2005), Mean LnP(K) (Pritchard et al. 2000) and MedMeaK (Puechmaille 2016). CLUMPAK output files rendered STRUCTURE bar plots illustrating membership of individuals to inferred genomic clusters. Discriminant analysis of principal components (DAPC), a multivariant method to infer the number of clusters within a group of genetically related individuals, was employed as a complementary approach to disclose the structure in the studied samples. The Adegenet package function ‘dapc’ in RStudio was used (Jombart and Ahmed 2011). A principal component analysis (PCA) from the matrix of genotypes was performed and then, a selected number of principal components (PCs) used as input for linear discriminant analysis (LDA). To determine the optimal number of PCs for LDA, a cross‐validation process was implemented, and the PCs associated with the lowest Root Mean Square Error (RMSE) retained. Furthermore, DAPCs that preserved at least 90% of the cumulative data variance were subject to evaluation.

### 2.5 Gene Mining

For each locus, the Rsb score was calculated using the rehh package in R, and the candidate genomic regions under divergent selection were identified. Genes within those genomic regions were retrieved from the *R*. *decussatus* genome and further inspected to look for candidate genes related to resistance/tolerance to *P. olsenii*, considering their immune function and the previous functional information in response to *P. olsenii* infections in *R*. *decussatus* (Estêvão et al. 2023) and *R*. *philipinarum* (Hasanuzzaman et al. 2018). Gene Ontology (GO) enrichment on the whole gene list within windows was performed with GOfuncR (https://bioconductor.org/packages/release/bioc/html/GOfuncR.html) using gene IDs of the annotated genome, taking as reference the whole transcriptome of *R*. *decussatus*.

## 3 Results

### 3.1 Genome Assembly

**FIGURE 2:**
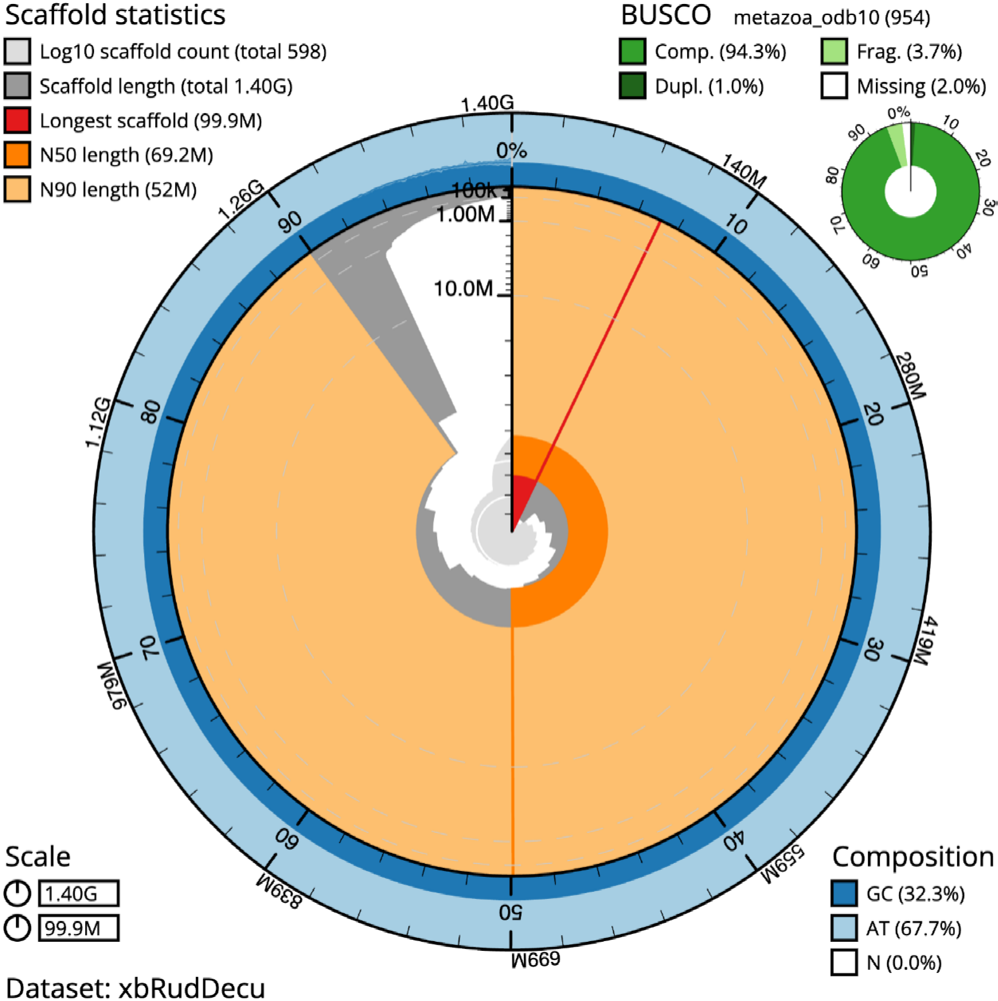
Snail plot showing contiguity and quality of *R*. *decussatus* genome assembly.

A total of 159.23 Gb was obtained for long‐read ONT sequencing (113×), 2926.26 Gb for 150 bp PE Illumina sequencing (191×) and 542.25 Gb for OmniC sequencing (386×). The genome assembly of *R*. *decussatus* comprised 1677 contigs with a contig N50 of 1.868 Mb for a total assembly size of 1406 Mb (Table 2). This assembly was rather fragmented, displaying consensus quality (QV = 39.13) and k‐mer (83.9%) completeness. After scaffolding with Hi‐C, contigs were assembled in 598 scaffolds, the largest 19 superscaffolds corresponding to the haploid chromosome number of the species (*n* = 19) and comprising 1301 Mb (92.5% of the total assembly) (Figure 2 and Table S1). Furthermore, another 90 scaffolds comprising 419 Mb could be placed but not mapped in one of the 19 superscaffolds (95.4% total assembly). The remaining 489 unplaced scaffolds comprised 56 Mb, ranging in length from 1000 bp to 533,292 bp. Genome completeness as assessed by BUSCO using the metazoa_odb10 lineage, was 94.3% complete (C:94.5%[S:93.5%, D:1.0%], F:3.7%, M:1.8%, n:954), in the upper range reported for bivalve assemblies in the last 5 years (Ran et al. 2019; Gundappa et al. 2022; Peñaloza et al. 2021; Boutet et al. 2022).

**TABLE 2:** Statistics of the chromosome‐level genome assembly of *R*. *decussatus*.

Contig N50 (bp)	1,867,564
Contig N90 (bp)	378,298
Max contig length (bp)	9,656,817
Mean contig len (bp)	838,478
Assembly span (bp)	1406,128,519
No. contigs	1677
No. gaps	316
GC content	32.34%
QV	39.13
kmer‐completeness	83.9%

### 3.2 Genome Annotation

In total, 38,276 protein‐coding genes that produce 54,530 transcripts (1.42 transcripts per gene) were annotated and encoded for 49,539 unique protein products (Table 3 and Table S2). Functional labels for 8.5% of the annotated proteins could be assigned (Table S3). The annotated transcripts contain 8.5 exons on average, with 86% being multi‐exonic. In addition, 13,056 non‐coding transcripts were annotated, of which 11,425 and 1631 corresponded to long and short non‐coding RNA genes, respectively.

**TABLE 3:** Annotation statistics of the *R*. *decussatus* genome.

Number of protein‐coding genes	38,276
Median gene length (bp)	7087
Number of transcripts	54,530
Number of unique protein products	49,539
Number of exons	283,949
Median UTR length (bp)	1585
Median intron length (bp)	811
Exons/transcript	8.49
Transcripts/gene	1.42
Multi‐exonic transcripts	86%
Gene density (gene/Mb)	27.38

### 3.3 SNP Calling

**FIGURE 3:**
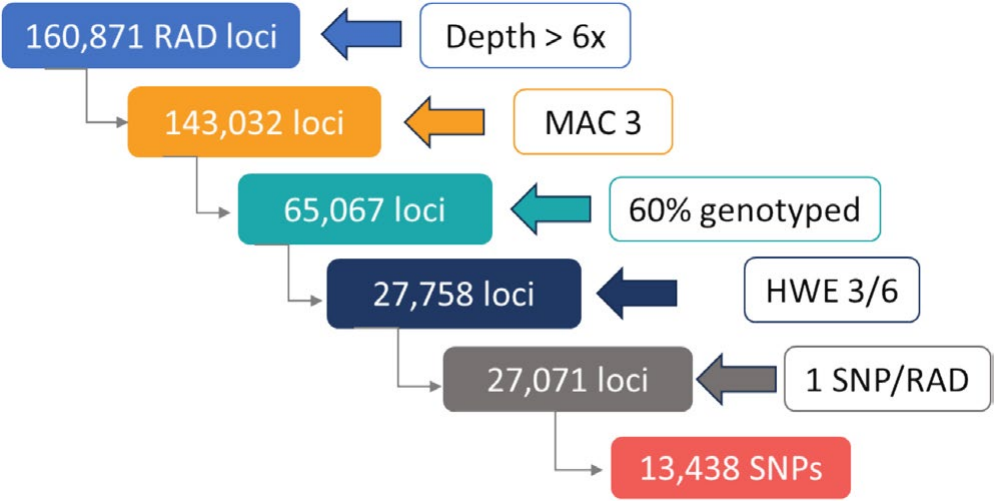
Bioinformatic filtering pipeline followed for SNP calling in *R*. *decussatus*.

From a total of 994.5 million reads obtained by 2bRAD‐Seq in the 215 individuals studied, 81.3% were retained after quality filtering. On average 46% of reads aligned to unique sites in the *R*. *decussatus* genome, representing 3.5 M reads per individual (ranging from 90 K to 4.3 M reads), most being discarded due to multiple alignments (34%) and only 1.4% did not aligned to the assembled genome. The gstacks module, using the marukilow model applied to all samples yielded 160,871 RAD loci. After filtering, a total of 13,438 SNPs were retained, which constituted the common catalogue for analyses in the six studied shellfish beds (Figure 1). The main filtering steps were “loci with MAC ≥ 3” (54.5%), “genotyped in 60% individuals” (57.4%) and “keeping only one SNP per RAD” (50.4%) (Figure 3).

### 3.4 Outlier Identification

Outliers and genomic regions under divergent selection were explored with the Rbs method of EHH software considering the main goals of the study: (i) to obtain a preliminary picture of the genetic structure of *R*. *decussatus* also considering outliers for divergent selection in Atlantic and Mediterranean shellfish beds; (ii) to identify candidate genes and markers associated with resistance/tolerance to *P. olsenii* by comparing groups of individuals classified by infection level (low, moderate, heavy) across shellfish beds with similar infection profiles. From outlier information, the following SNP datasets were used to analyze genetic diversity and population structure: (i) the whole SNP dataset; (ii) SNP dataset under divergent selection obtained from the geographic scenarios explored; (iii) neutral SNPs obtained by excluding all outliers (geographical and perkinsosis infection) from the whole SNP dataset. For the analyses, both Venice samples were pooled (60 individuals), after confirming no significant genetic differentiation between them (see below).

The geographical analysis revealed 394 SNPs under divergent selection in the 15a pair‐wise comparisons tested (ATL (3), MED (3), ATL vs. MED (9)) (Tables S4–, S7). Many outliers were specific to each pairwise comparison in all scenarios, and the number of outliers detected was higher in the ATL vs. MED (176, Table S6) and the ATL (145, Table S4) scenarios than in the MED scenario (105, Table S5).

When exploring signals of selection for resistance/tolerance to *P. olsenii*, we detected a total of 343 outliers by comparing the level of infection (low: L0 + L1; moderate: L2 + L3; heavy: L4 + L5) using two population pairs showing a similar infection profile (ATL scenario: ALG and PO; MED scenario: VEN and TU) (Tables S8–, S10); among them, 191 were detected in the ATL scenario (Table S8), 158 in the MED scenario (Table S9), and 90 were shared between both scenarios or lay within the same genomic window showing signals of divergent selection (< 1 Mb distance; 29 genomic windows, Table S10). Genes within these genomic windows were inspected for their putative association with immune response and with previous studies on perkinsosis considering their mapping on the most consistent genomic windows.

### 3.5 Genetic Diversity

Average observed (Ho) and expected (He) heterozygosity and allelic richness (Ar) were first estimated with the whole SNP dataset. All estimators showed significantly higher genetic diversity in the Mediterranean than in the Atlantic region (average Ar: 1.797 vs. 1.604; He: 0.242 vs. 0.173; Mann–Whitney test *p* < 0.001 in all cases) (Table 4A). VEN samples were by far the most diverse samples among all studied (Ar = 1.854; He = 0.233). All sampling locations conformed to HWE (*p* > 0.05). Similar observations were obtained when using the neutral dataset (data not shown).

**TABLE 4:** Genetic diversity and intrapopulation fixation index (F_IS_) of *R. decussatus* populations with: (A) whole dataset; (B) geographical outliers for divergent selection.

(A)				
	Ar	He	Ho	FIS
ALG	1.595	0.166	0.166	0.003
NO	1.606	0.17	0.167	0.021
PO	1.610	0.168	0.169	0.002
SAR	1.711	0.195	0.200	−0.008
TU	1.769	0.203	0.198	0.027
VEN	1.854	0.233	0.228	0.037
Atlantic	1.604	0.173	0.166	0.009
Mediterranean	1.797	0.242	0.211	−0.008

(B)				
	Ar	Ho	He	FIS
ALG	1.660	0.156	0.157	0.012
NO	1.715	0.199	0.209	0.044
PO	1.735	0.219	0.226	0.017
SAR	1.850	0.303	0.297	−0.005
TU	1.885	0.264	0.276	0.038
VEN	1.967	0.346	0.365	0.051
Atlantic	1.797	0.191	0.212	0.024
Mediterranean	1.993	0.315	0.372	0.028

When using the geographical outlier panel for divergence selection (Table 4B), genetic diversity was significantly higher for all estimators in the Mediterranean than in the Atlantic region, as observed with the whole SNP dataset; additionally, genetic diversity was significantly higher for the outlier than for the neutral dataset both in the Atlantic (average Ar: 1.797 vs. 1.615; He: 0.212 vs. 0.170; Mann–Whitney test *p* < 0.05) and the Mediterranean (average Ar: 1.993 vs. 1.797; He: 0.372 vs. 0.237; Mann–Whitney test *p* < 0.05) regions.

### 3.6 Genetic Structure

The two samples from Venice lagoon (VE19 and VE22) showed no genetic differentiation between them (F_ST_ = 0.002; *p* > 0.05) and accordingly, they were pooled into a single sample of 60 individuals. The global F_ST_ for all samples using the whole SNP dataset was 0.180 (*p* < 0.001). The most pronounced genetic differentiation was detected between Atlantic and Mediterranean samples (average pairwise F_ST_ = 0.224; Table 5A). No significant genetic differentiation was detected between NO and PO (*p* > 0.05 after Bonferroni correction), while ALG and TU were the most differentiated populations (F_ST_ = 0.356; *p* = 0). Higher differentiation was detected within the Mediterranean Sea than within the Atlantic Ocean (average pairwise F_ST_ = 0.169 vs. 0.031, respectively). Pairwise F_ST_ comparisons using the neutral SNP panel showed very similar results (data not shown).

**TABLE 5:** Pairwise F_ST_ values between populations of *R. decussatus* with (A) whole SNP dataset; (B) geographic divergent selection outliers. Below and above the diagonal F_ST_ and probability values, respectively.

(A)						
	ALG	NO	PO	SAR	TU	VEN
ALG	—	*	*	*	*	*
NO	0.045	—	0.032	*	*	*
PO	0.048	**0.001**	—	*	*	*
SAR	0.158	0.152	0.150	—	*	*
TU	0.355	0.349	0.349	0.2841	—	*
VEN	0.173	0.168	0.166	0.085	0.137	—

(B)						
	ALG	NO	PO	SAR	TU	VEN
ALG	—	*	*	*	*	*
NO	0.129	—	0.293	*	*	*
PO	0.127	**0.010**	—	*	*	*
SAR	0.272	0.224	0.206	—	*	*
TU	0.485	0.434	0.416	0.346	—	*
VEN	0.266	0.224	0.209	0.123	0.135	—

*Note:* In bold highlighted no significant F_ST_ values after Bonferroni correction (*p* < 0.003).

* *p* < 0.001.

A much higher differentiation was found when using the geographic divergent outlier SNPs, although following a similar pattern to that observed with the whole and neutral datasets (Table 5B). No significant differentiation was detected between the two close Spanish populations (NO and PO) but figures increased abruptly in all other cases with F_ST_ > 0.2 in all Atlantic vs. Mediterranean comparisons (average F_ST_ = 0.318). TU displayed the highest differentiation with Atlantic populations (F_ST_ > 0.4), but also important with the other Mediterranean populations, especially SAR (F_ST_ = 0.346).

**FIGURE 4:**
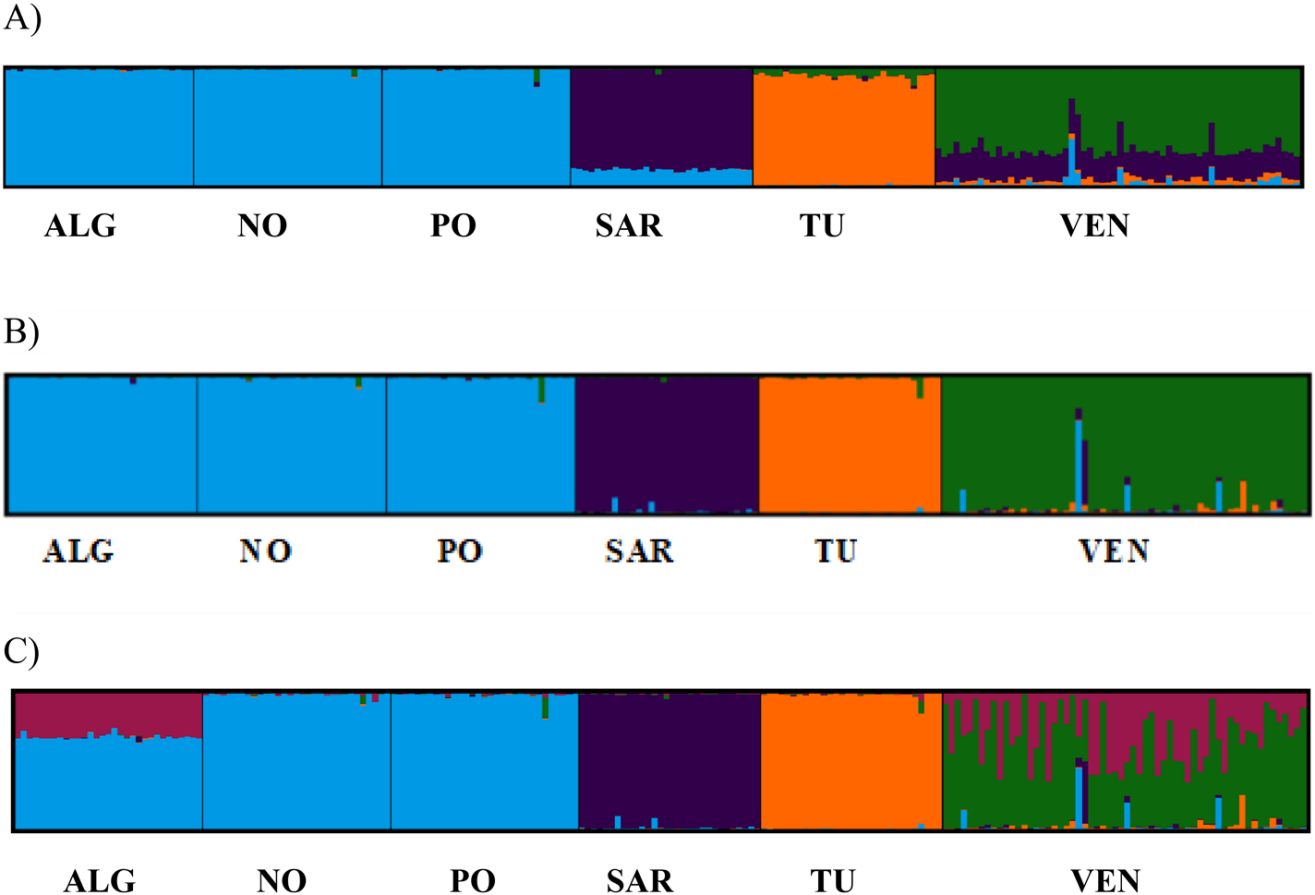
STRUCTURE analysis in *R*. *decussatus* with (A) the whole panel for *K* = 4; (B) and (C) geographical outlier panel for *K* = 4 and *K* = 5, respectively.

The clustering method of STRUCTURE was run for *K* values ranging from 1 to 7 (number of samples plus 1; Figure S4) using deltaK and Mean LnP(K) estimators; *K* = 4 was the optimal number of clusters with the whole and neutral SNP datasets. The results revealed a single cluster comprising the three Atlantic populations, while the three Mediterranean samples, TU, SAR and VEN, constituted separate entities, with SAR showing a certain Atlantic component and VEN some admixture from different clusters (Figure 4A).

When employing the divergent geographic outlier panel, the results were slightly different depending on the method used, the most likely number of clusters (K) being either 4 or 5 (Figure 4B,C and Figure S5). The three Atlantic populations constituted a single cluster for *K* = 4, while the three Mediterranean populations clearly constituted separate units, more than with the whole SNP dataset. However, a notable differentiation of the southernmost population (ALG) was detected for K = 5 in the Atlantic, and within the Mediterranean Sea, the Venice Lagoon (VE) showed an admixed composition mainly from ALG but also, to a minor extent, from the other clusters.

The discriminant analyses of principal components (DAPC) complemented and confirmed the results observed with STRUCTURE (Figure S6). Atlantic samples were tightly clustered with the whole SNP dataset, while the Mediterranean samples were highly differentiated. Some nuances could be unveiled with the divergent outlier panel, which depicted the ALG slightly differentiated from the northern Atlantic NO and PO; additionally, VE was nearly equidistant from TU and SAR, but also from the Atlantic cluster.

### 3.7 Perkinsus Resistance/Tolerance SNP Outliers: Gene Mining

Broad‐sense heritability was estimated with the whole SNP and infection‐level dataset using the GCTA program (Yang et al. 2011). The moderate‐high heritability obtained (h^2^ = 0.579 ± 0.180), highly significant despite the error associated with the wild scenario inspected (*p* < 0.001), supports a genetic component underlying resistance/tolerance to *P. olsenii*. A total of 90 outliers related to the *Perkinsus* resistance/tolerance were shared or mapped in the same genomic window (< 1 Mb) when comparing the ATL (ALG and PO) and MED (VEN and TU) infection‐level approach (Table S10). The 29 genomic windows where the infection‐level outliers were located (43 Mb; 3.07% of *R*. *decussatus* genome) included 439 genes that were inspected to identify candidates related to perkinsosis resistance/tolerance (Table S11). No functional enrichment GO terms were identified in this set of genes using GOfuncR, despite the very important immune‐related gene families included, such as complement C1q, E3 ubiquitin‐protein ligase, glutathione peroxidase, MAM and LDL‐receptor class A, lysozyme, cytochrome P450, lymphocyte antigen, N‐acetylglucosamine‐1‐phosphotransferase, peptidoglycan recognition protein, peroxidase, proteasome, prostaglandin E2 receptor, serine/threonine‐protein kinase, toll‐like receptor, and tripartite motif‐containing protein, among others (Table S11B). The poor functional annotation of *R*. *decussatus* genome (8.5%; Table S3), a usual feature of mollusc genomes (Liu et al. 2021), likely underlies this outcome. Then, we compared our list of 439 genes with those from the functional studies performed by Estêvão et al. (2023; proteomic) in *R*. *decussatus* and by Hasanuzzaman et al. (2018; transcriptomic) in the congeneric species *R*. *philippinarum* in response to *P. olsenii* (Table S12). Among them, we could identify five genes related to iron storage, cytoskeleton organization, proteases, and energy balance with identical or similar annotation to the list of 32 proteins differentially expressed when comparing heavily infected vs. non‐infected clams by Estêvão et al. (2023). Specifically, phosphoenolpyruvate carboxykinase [GTP] and proteasome subunit alpha type were also detected as differentially expressed (DEG) in response to infection in the wild and controlled laboratory conditions by Hasanuzzaman et al. (2018). Furthermore, we could identify 12 genes with identical annotation to the DEGs reported by Hasanuzzaman et al. (2018) (Table S12). Four of them, complement C1q‐like protein 4, cytochrome P450 2A6, tyrosine‐protein kinase, and ubiquitin‐conjugating enzyme E2 U, were considered crucial in the response of *R*. *philippinarum* to *P. olsenii* by these authors. Other 15 DEGs showed very similar annotation or pertained to the same gene family, and among these, caprin‐1, low‐density lipoprotein receptor‐related protein 2, palmitoyltransferase B, and universal stress protein A. R, were also considered critical by Hasanuzzaman et al. (2018) in the response to *Perkinsus*.

## 4 Discussion

The increasing prevalence of *P. olsenii* poses a significant challenge to aquaculture, resulting in extensive mortalities and adverse impacts on clam health, which translate into economic losses and disruption of marine ecosystem balance. Perkinsosis shows variable incidence throughout *R. decussatus* shellfish beds, from the Atlantic Ocean to the Mediterranean Sea, embracing east and west Mediterranean beds. Thus, the first goal of the present study was to obtain information on the genetic diversity and structure of the species across the whole distribution range, applying for the first time SNP markers covering the whole genome at medium density (13,438 SNPs; ~1 SNP/100 kb), taking as reference a new chromosome‐level genome here assembled. This enabled us to contrast previous information obtained on the genetic structure of the species with a low number of mtDNA, isozyme, microsatellite, and SNP markers (Cordero et al. 2014; Arias‐Pérez et al. 2016; Cruz et al. 2020) and to identify suggestive outlier loci associated with divergent selection across the grooved carpet clam distribution to be thoroughly explored in the future.

### 4.1 Genetic Structure

Genetic diversity was significantly higher in Mediterranean than in Atlantic populations, as previously reported (Cordero et al. 2014; Arias‐Pérez et al. 2016). Consistent geographic differentiation was detected between Atlantic and Mediterranean populations using the whole or the neutral SNP datasets (average pairwise F_ST_ = 0.224 and 0.193, respectively), in the range reported in other studies using nuclear RFLPs and microsatellites (Arias‐Pérez et al. 2016; Saavedra and Cordero 2024). A much lower differentiation was found between the Atlantic samples (average pairwise F_ST_ = 0.031), which constituted a single cluster with STRUCTURE and DAPC analyses, than between the Mediterranean ones (F_ST_ = 0.169), which essentially represented three differentiated clusters. This observation could be partially related to the different geographic extension surveyed in the Atlantic Ocean and the Mediterranean Sea, but it has also been reported in other aquatic species across a similar distribution range (Maroso et al. 2019; Saavedra and Cordero 2024). Our data suggest a higher isolation of Mediterranean populations either by a more complex current/front marine pattern or by less efficient larval dispersion mechanisms that could be operating in an inner sea, and/or reflecting long‐term evolutionary processes (Bierne et al. 2011). Within the Mediterranean Sea, the TU and SAR samples, located in the eastern and western regions, displayed the greatest differentiation (F_ST_ = 0.283), and as previously suggested with mtDNA markers (Arias‐Pérez et al. 2016), the SAR population was closer to the Atlantic samples than to the other Mediterranean shellfish beds. All data soundly point towards a mixed origin of the population from Venice lagoon that showed higher genetic diversity and admixed constitution, including a certain component from the Atlantic which agrees with anectodical evidence of restocking in that area using clams of various origins (L. Bargelloni, personal communication). Genetic differentiation greatly increased when exploring outlier loci under divergent selection, particularly when comparing Atlantic and Mediterranean samples (average F_ST_ = 0.318), suggesting adaptation to the very different environmental conditions between both body waters and/or reflecting long‐term evolutionary processes (Bierne et al. 2011). Despite the Atlantic Ocean showed lower differentiation with divergent outliers than the Mediterranean Sea (average F_ST_ = 0.089 vs. 0.201), the percentage with respect to the neutral dataset increased much more in the Atlantic Ocean (average F_ST_ = 0.089 vs. 0.028) than in the Mediterranean Sea (0.201 vs. 0.168), which could suggest a more heterogeneous environment in the Atlantic Ocean. In fact, this is also supported by the higher number of divergent outliers detected within the Atlantic than within the Mediterranean area (156 vs. 116). The results, although based on a limited sampling collection, support previous observations, but provide new insights on genetic diversity and structure across the *R*. *decussatus* distribution range, emphasizing the important differentiation of a species living in highly diverse environmental conditions that should be considered both for broodstock foundation as well as for genetic‐environmental interactions in production areas.

### 4.2 Genetic and Environmental Factors Underlying Perkinsosis

*Perkinsus* infection of clams exhibits variable outcomes depending on environmental conditions, especially temperature and salinity, which have been reported to play an important role in parasite prevalence (Villalba et al. 2004; Ruano et al. 2015). Elevated water temperatures have been linked to increased *Perkinsus* prevalence, and variations in salinity levels have been observed to influence the susceptibility of *R. decussatus* to *P. olsenii*, emphasizing the need for a comprehensive examination of these factors (Casas and Villalba 2012). However, high prevalence of the parasite has been detected both in Atlantic and Mediterranean populations, despite the temperature and salinity differences in both regions (this study; Ruano et al. 2015). Parasite prevalence may also be related to the genetic constitution of populations, and the genetic differentiation observed between them in our study might reflect differences in resistance/tolerance to perkinsosis. Noia estuary was the only perkinsosis‐free area among the populations studied, but this appears to be related to the particular current patterns in NW Spain, since this estuary has proved to be also free of other emergent parasites in bivalves, such as *Marteilia cochillia* in common cockle ( *Cerastoderma edule* ) (Pampín et al. 2023; Villalba et al. 2023). Within the Mediterranean Sea, the prevalence was much lower in SAR, which could be related to its insular condition or to less impact of clam species transference from other areas. Despite the other two Atlantic (PO, ALG) and Mediterranean (VEN, TU) samples showing nearly 100% prevalence, the average infection level was significantly lower in the Atlantic than in the Mediterranean populations. Indeed, perkinsosis incidence has been associated with temperature and salinity. However, a higher parasite pressure should determine changes in the genetic constitution of populations associated either with resistance or tolerance (or both), especially considering the significant heritability estimated in our study, and in fact, genetic divergence has been detected for specific genomic regions including immune‐related genes in the two scenarios studied (ALG & PO; VEN and TU). Furthermore, although common genomic regions were detected in the two scenarios, specific signals were also detected within each scenario, suggesting an adjusted response depending on parasite diversity, environmental factors, or their interaction.

### 4.3 Genetic Markers and Candidate Genes for Resistance/Tolerance to *P. olsenii*

Resistance, tolerance, and resilience are terms featuring host–parasite interactions that have been largely discussed in the literature (Holbrook et al. 2021; Paraskevopoulou et al. 2022; Råberg et al. 2008). While resistance refers to the capacity of the host to avoid the parasite's entrance or to eliminate it once inside, tolerance is related to the ability of the host to maintain a certain level of health/fitness and to the ability to neutralise the virulence of the parasite. On the other hand, resilience defines the capacity of recovery from a parasite infection. Discrimination of the different components is more affordable under controlled experimental conditions, so in the wild samples of our study we could hardly discriminate between them, especially resilience. Furthermore, it is also possible that individuals with no infection have recovered from a previous infection, and this cannot be distinguished from resistant individuals to infection. Bearing in mind these considerations, we decided with all cautions to use only the combination of resistance/tolerance to interpret the association of markers with the different infection levels, despite some features that could be associated with resistance (or recovery) and tolerance.

Most samples in the present study have probably been in contact with the parasite at any time in their life considering the long infection records of shellfish beds (Ruano et al. 2015) and the age of the individuals collected (adults), resulting in the high prevalence observed (close to 100% in most cases, excluding NO, naïve). However, as outlined before, perkinsosis pressure probably differs across the shellfish beds studied and, accordingly, the genetic response underlying the resistance or tolerance to perkinsosis. Thus, despite detecting signals of selection for perkinsosis in the wild is more complex and possibly confounded with other environmental factors associated with geography, it offers the opportunity to check broader scenarios to be interpreted for a more comprehensive understanding of this parasitosis. On the other hand, challenges in laboratory conditions enable a much better control of environmental factors, and thus an increasing statistical power to detect QTL associated with resistance/tolerance, but they do not consider the environmental variation in the wild, where clam production takes place.

In our study, the broad sense heritability estimated suggests a significant genetic component underlying resistance/tolerance (h^2^ = 0.579 ± 0.180). We could identify a set of 90 SNPs located in 29 genomic windows (43 Mb, 3.07% genome) related to divergent selection for resistance/tolerance to perkinsosis shared in the two infection‐level scenarios (low, moderate, heavy), Atlantic (ALG and PO) and Mediterranean (VEN, TU), mapping on the assembled chromosomes (supercaffolds) of *R*. *decussatus*. The list of 439 genes within those genomic windows enabled us to identify candidates related to resistance/tolerance and to compare them with those previously reported using functional approaches in response to *P. olsenii* in *R*. *decussatus* (Estêvão et al. 2023) and *R*. *philippinarum* (Hasanuzzaman et al. 2018). It should be noted that the study by Hasanuzzaman et al. (2018) involved the comparison of transcriptomic response between different levels of infection in the wild, a similar experimental scenario to our infection‐level comparison. Among the most relevant genes here identified in the two infection‐level scenarios inspected, the phosphoenolpyruvate carboxykinase [GTP], a key enzyme related to energy balance through gluconeogenesis but also involved in bacteria recognition and elimination (Lv et al. 2017), and several proteasome‐related genes, involved in cell proliferation and differentiation, replication of protozoan parasites, and stress response (Fernández‐Boo et al., 2014; Portilho et al. 2019), were also differentially expressed in the two functional studies outlined before. The same occurred with several DEGs detected in the transcriptome response of *R*. *philippinarum* to *P. olsenii* in the wild, some of them considered critical in host–parasite interaction by Hasanuzzaman et al. (2018). Among them were complement C1q‐like protein 4 and cytochrome P450 2A6, involved in immune and stress response (Lüchmann et al. 2015; Nie et al. 2016; Wang et al. 2024); tyrosine‐protein kinase, ubiquitin‐conjugating enzyme E2 U, and caprin 1, involved in apoptosis and cell proliferation pathways (Prado‐Alvarez et al. 2009; Leite et al. 2013; Romero et al. 2015). On the other hand, low‐density lipoprotein receptor‐related protein 2 and palmitoyltransferase related to lipid homeostasis were also identified as potential biomarkers by Hasanuzzaman et al. (2018). Finally, several genes pertaining to relevant immune and stress‐related families in molluscs, such as E3 ubiquitin, ras‐related proteins, heat shock proteins, serine/threonine proteins, universal stress proteins, and vacuolar protein sorting‐associated protein (Huang et al. 2015; Cheng et al. 2016; Guo et al. 2018; Ronza et al. 2018; Lou et al. 2020; Smits et al. 2020; de la Ballina et al. 2021), identified in our study and by Hasanuzzaman et al. (2018), will deserve further investigation to understand the response of the grooved carpet shell clam to perkinsosis.

## 5 Conclusions

The first chromosome‐level genome of *R. decussatus* was assembled and used as a reference for a population genomics study throughout the distribution range of the species. We could consistently genotype > 13,000 SNPs using 2b‐RADseq for the first genomic screening of *R*. *decussatus* involving Mediterranean and Atlantic regions. Our data confirmed the main features of the genetic structure previously reported but also made it possible to identify a subset of SNPs related to divergent selection putatively associated with environmental factors to be further explored. The comparison of perkinsosis infection across populations with similar infection profiles allowed the consistent identification of 90 markers and associated genomic windows related to divergent selection for resistance/tolerance to *P. olsenii*, including candidate genes previously considered critical in the response of infected clams in functional studies in the wild. This information should be considered for understanding the genetic basis of this parasitosis, which should be helpful to devise strategies for its control and to establish founder populations in hatcheries for improved seed resistance.

## Ethics Statement

The authors have nothing to report.

## Conflicts of Interest

The authors declare no conflicts of interest.

## Supporting information


Figure S1.
Figure S2.

Figure S3.

Figure S4.

Figure S5.

Figure S6.

Data S1.

Table S1.

Table S2.

Table S3.

Table S4.

Table S5.

Table S6.

Table S7.

Table S8.

Table S9.

Table S10.

Table S11.
Table S12.

## Data Availability

Genotyping data of the whole sample dataset organized by geographical origin and level of infection criteria have been uploaded to Dryad (https://doi.org/10.5061/dryad.wh70rxx0r).
