# Supplementary material for: Signatures of Selection for Resistance/Tolerance to Perkinsus olseni in Grooved Carpet Shell Clam (Ruditapes decussatus) Using a Population Genomics Approach
Source: Evol Appl. 2025 May 13;18(5):e70106. doi: 10.1111/eva.70106 (PMC12070250; doi:10.1111/eva.70106)
Supplement: Supplementary file 1 — Figure S1. Figure S2. [file EVA-18-e70106-s006.pptx]

## Slide 1
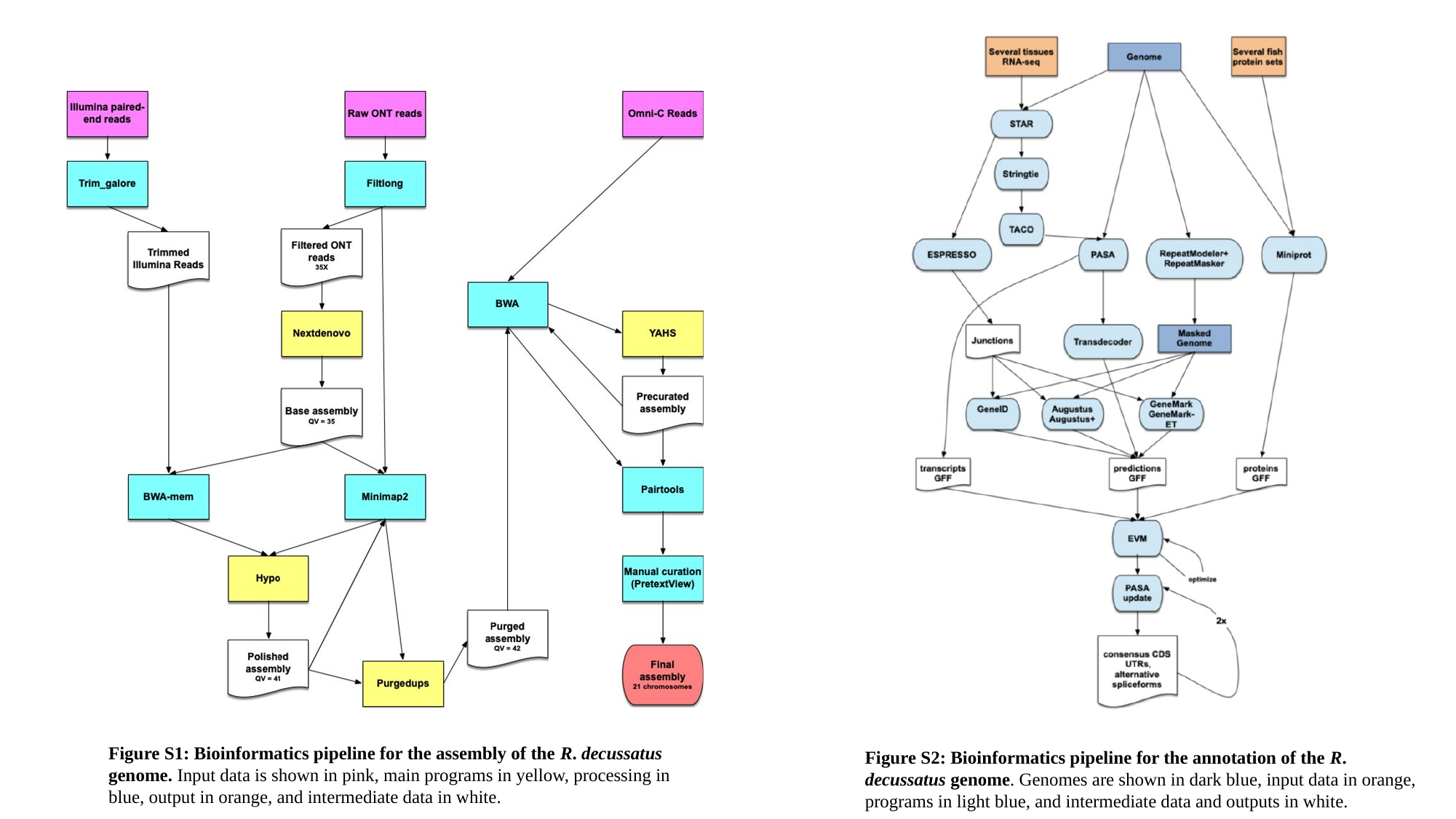

Figure S2: Bioinformatics pipeline for the annotation of the R. decussatus genome. Genomes are shown in dark blue, input data in orange, programs in light blue, and intermediate data and outputs in white.
Figure S1: Bioinformatics pipeline for the assembly of the R. decussatus genome. Input data is shown in pink, main programs in yellow, processing in blue, output in orange, and intermediate data in white.
