# Supplementary material for: Signatures of Selection for Resistance/Tolerance to Perkinsus olseni in Grooved Carpet Shell Clam (Ruditapes decussatus) Using a Population Genomics Approach
Source: Evol Appl. 2025 May 13;18(5):e70106. doi: 10.1111/eva.70106 (PMC12070250; doi:10.1111/eva.70106)
Supplement: Supplementary file 2 — Figure S3. [file EVA-18-e70106-s003.pptx]

## Slide 1
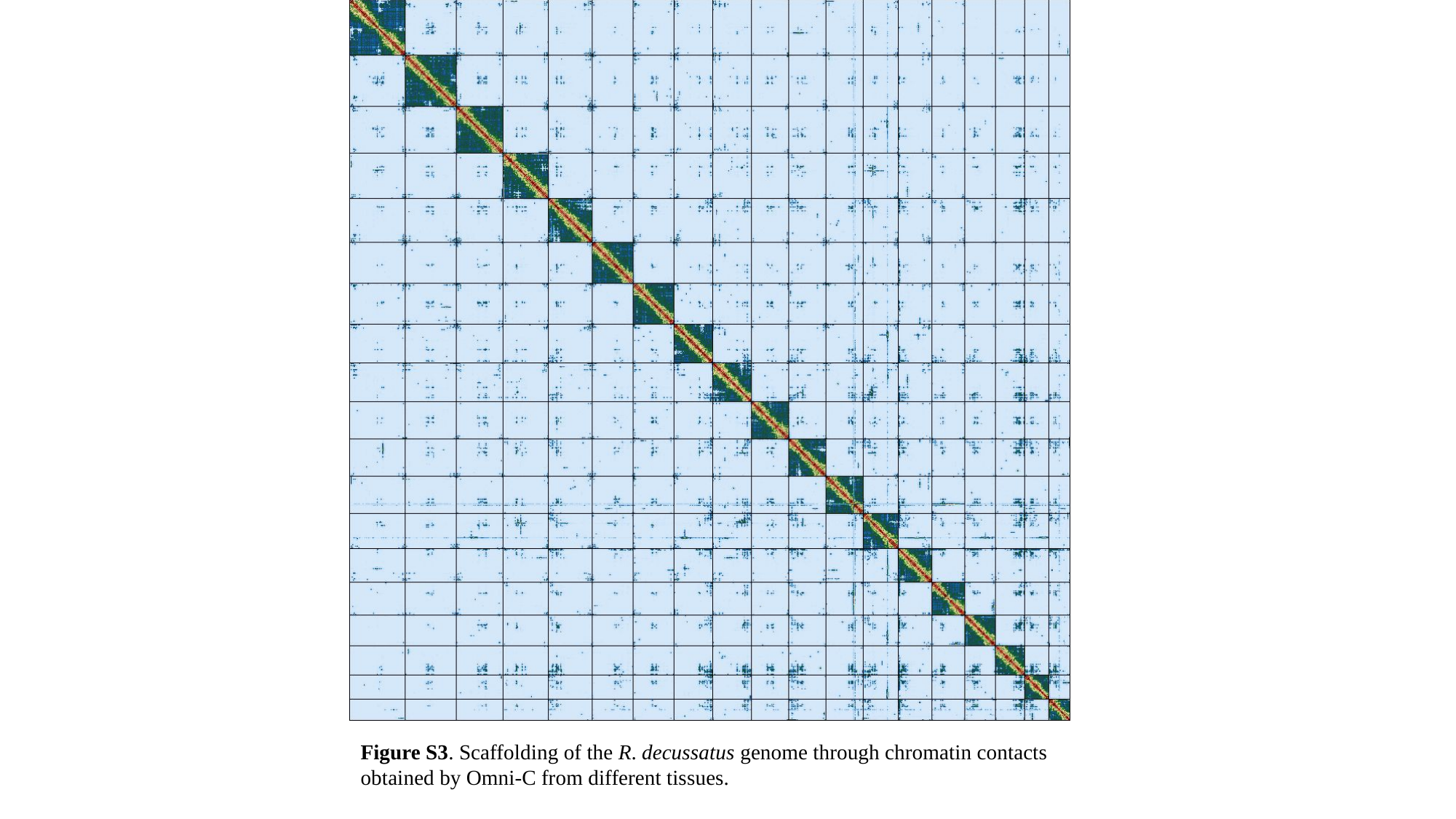

Figure S3. Scaffolding of the R. decussatus genome through chromatin contacts obtained by Omni-C from different tissues.
