# Supplementary figures and images for: Signatures of Selection for Resistance/Tolerance to Perkinsus olseni in Grooved Carpet Shell Clam (Ruditapes decussatus) Using a Population Genomics Approach
Source: Evol Appl. 2025 May 13;18(5):e70106. doi: 10.1111/eva.70106 (PMC12070250; doi:10.1111/eva.70106)

**Figure S4**: STRUCTURE analysis in *R*. *decussatus* with the geographic outlier SNP panel for K=1-8


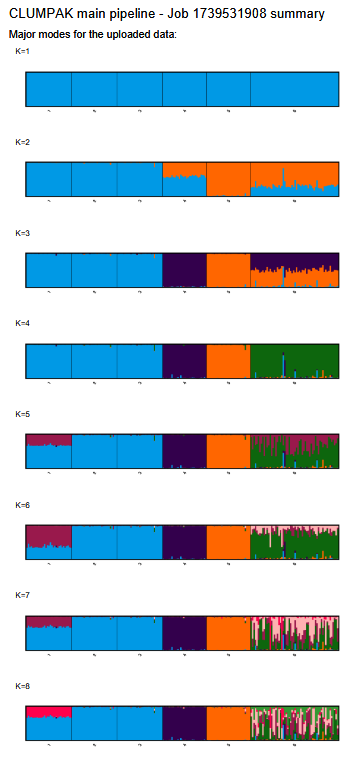

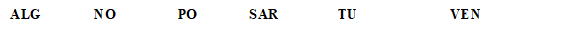

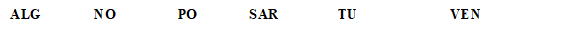

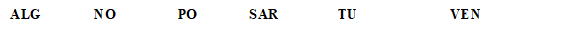

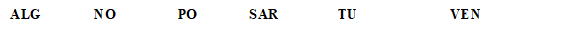

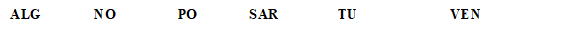

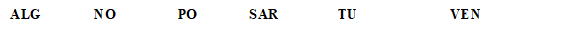

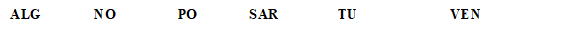

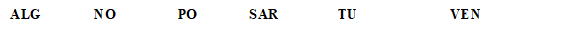

Supplement: Supplementary file 3 — Figure S4. [file EVA-18-e70106-s001.docx]

**Figure S5**: STRUCTURE analysis in *R*. *decussatus* with the geographic outlier SNP panel for K=1-8


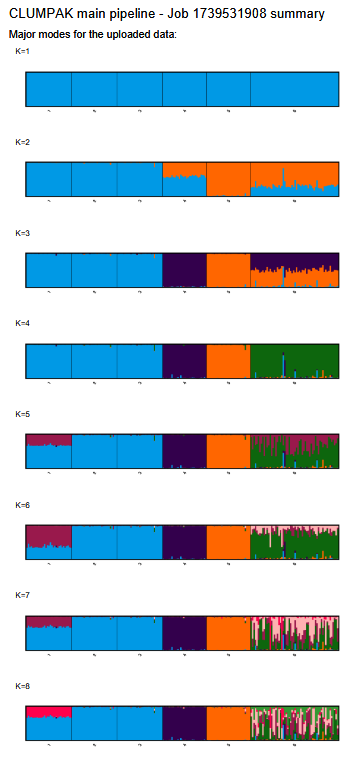

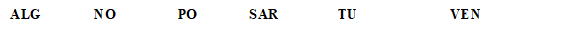

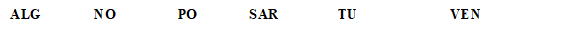

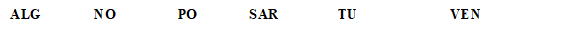

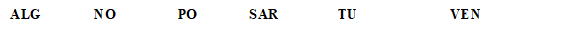

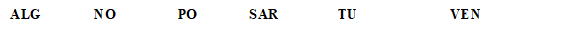

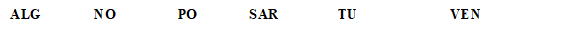

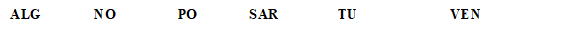

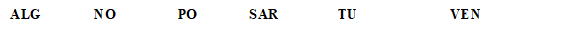

Supplement: Supplementary file 4 — Figure S5. [file EVA-18-e70106-s017.docx]
