# Supplementary material for: Signatures of Selection for Resistance/Tolerance to Perkinsus olseni in Grooved Carpet Shell Clam (Ruditapes decussatus) Using a Population Genomics Approach
Source: Evol Appl. 2025 May 13;18(5):e70106. doi: 10.1111/eva.70106 (PMC12070250; doi:10.1111/eva.70106)
Supplement: Supplementary file 5 — Figure S6. [file EVA-18-e70106-s009.pptx]

## Slide 1
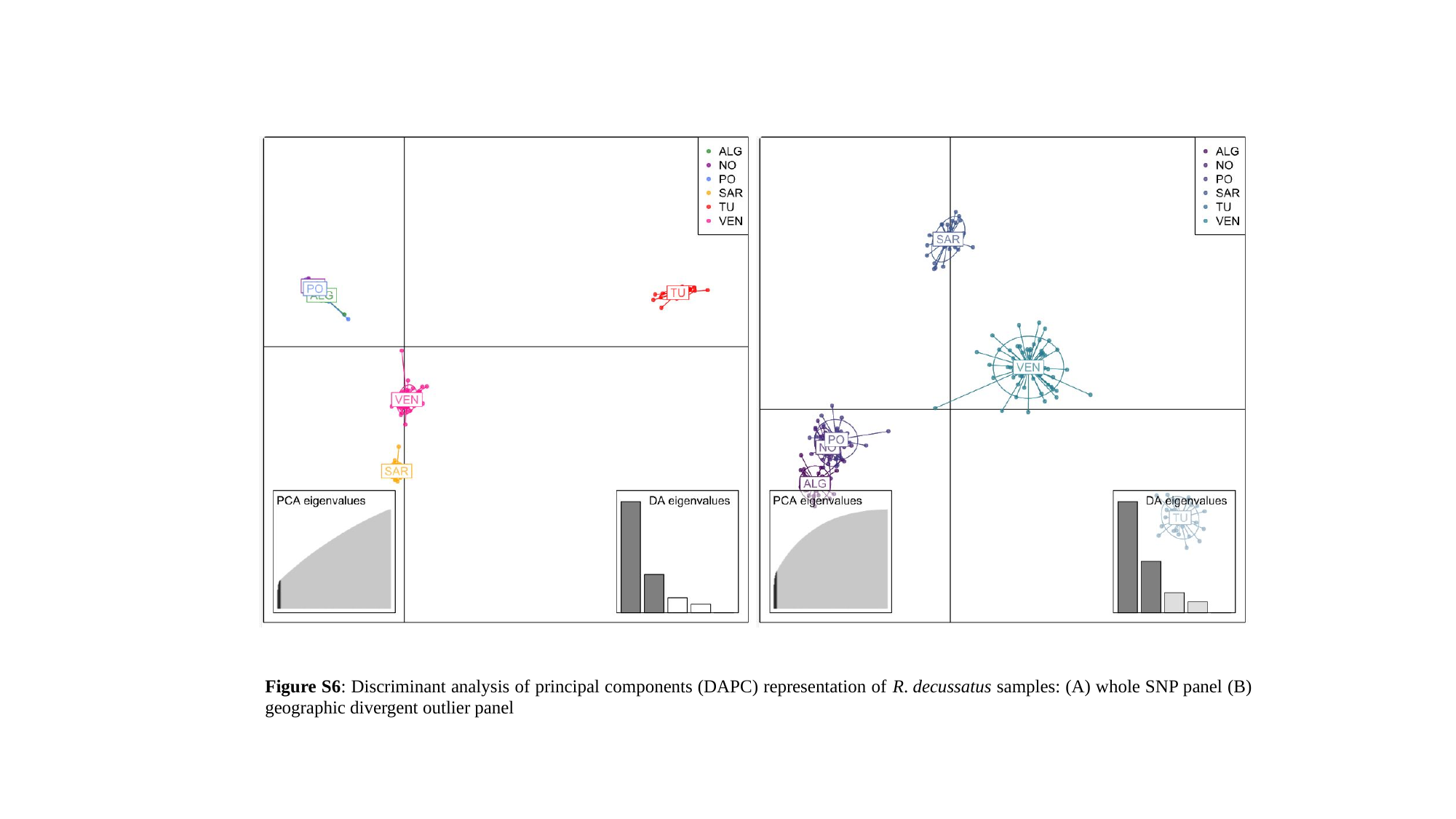

Figure S6: Discriminant analysis of principal components (DAPC) representation of R. decussatus samples: (A) whole SNP panel (B) geographic divergent outlier panel
