# Supplementary material for: Signatures of Selection for Resistance/Tolerance to Perkinsus olseni in Grooved Carpet Shell Clam (Ruditapes decussatus) Using a Population Genomics Approach
Source: Evol Appl. 2025 May 13;18(5):e70106. doi: 10.1111/eva.70106 (PMC12070250; doi:10.1111/eva.70106)
Supplement: Supplementary file 6 — Data S1. [file EVA-18-e70106-s015.docx]

**SUPPLEMENTARY METHODS**

*Whole Genome Sequencing (WGS)*

Long-read sequencing libraries

4.0 μg of the DNA was DNA-repaired and DNA-end-repaired using NEBNext FFPE DNA Repair Mix (NEB) and the NEBNext UltraII End Repair/dA-Tailing Module NEB, respectively. Then, sequencing adaptor ligation, purification by 0.4X AMPure XP Beads and elution in Elution Buffer (SQK-LSK109) was accomplished. The sequencing runs were performed on GridION Mk1 (ONT) using a Flowcell R9.4.1 FLO-MIN106D (ONT) and the sequencing data was collected for 110 hours.

Short-read genome sequencing libraries

1.0 µg of genomic DNA was sheared on a Covaris™ LE220-Plus (Covaris) and size-selected for the fragment size of 220-550 bp with AMPure XP beads (Agencourt, Beckman Coulter). The genomic DNA fragments were then end-repaired and adenylated. Next, compatible adaptors for Illumina platforms with unique dual indexes including unique molecular identifiers (Integrated DNA Technologies) were ligated.

Hi-C sequencing

Chromatin was fixed in place with formaldehyde (Sigma Aldrich), digested with DNase I and DNA extracted. DNA ends were repaired, and a biotinylated bridge adapter was ligated followed by proximity ligation of adapter-containing ends. After reverse crosslinking, the DNA was purified and followed by the preparation of Illumina-compatible paired-end sequencing libraries (omitting the fragmentation step). Biotinylated chimeric molecules were isolated using streptavidin beads before PCR enrichment of the library.

*Genome assembly*

Preprocessing and filtering of reads

Illumina reads were trimmed using Trim-galore v0.6.6 (with options *--gzip -q 20 --paired --retain_unpaired*) (<https://www.bioinformatics.babraham.ac.uk/projects/trim_galore/>) and the nanopore reads were filtered using FiltLong v0.2.0 (with options *--min_length* 5000 *--target_bases* 40,000,000,000) (FiltLong: <https://github.com/rrwick/Filtlong>). The filtering of nanopore data ensured having reads of at least 5 kb while optimizing for both length and higher mean base qualities, keeping 40 Gb (~ 65x coverage).

Filtered ONT reads were assembled with NextDenovo v2.4.0 (https://github.com/ Nextomics/NextDenovo) applying the options: minimap2_options_raw = -x ava-ont, minimap2_options_cns = -x ava-ont -k17 –w17 and seed_cutoff=10k. The resulting contigs were polished with Nextpolish v1.3.1 (Hu et al., 2020) using two rounds of long-read polishing and two rounds of short-read polishing.

The Omni-C reads were mapped to the assembly using BWA-MEM and pre-processed using the Dovetail pipeline (https://omni-c.readthedocs.io/en/latest/fastq_to_bam.html). The filtering of the alignments was done with the default minimum mapping quality of 40. After the removal of PCR duplicates, YaHS30 v1.1 was used for scaffolding with default parameters. Two rounds of assembly error correction were performed and made 15 breaks, followed by ten rounds of scaffolding from higher to lower resolution (10 Mb down to 10 Kb).

*Genome annotation*

RNA-Seq

The RNA-Seq libraries were prepared with KAPA Stranded mRNA-Seq Illumina® Platforms Kit (Roche). Briefly, 500 ng of total RNA was used for the poly-A fraction enrichment with oligo-dT magnetic beads, following the mRNA fragmentation protocol. The strand specificity was achieved during the second strand synthesis performed in the presence of dUTP instead of dTTP. The blunt-ended double stranded cDNA was 3´adenylated before Illumina platform compatible adaptors with unique dual indexes and unique molecular identifiers (Integrated DNA Technologies) were ligated.

Repetitive elements

Repeats present in the *R*. *decussatus* genome assembly were annotated with RepeatMasker v4-1-5-0 (http://www.repeatmasker.org) using the custom repeat library available for Mollusca. After excluding those repeats that were part of repetitive protein families (performing a BLAST search against UniProt) from the resulting library, RepeatMasker was run again with this new library to annotate the specific repeats. Next, *redmask* (<https://github.com/nextgenusfs/redmask>) was run on the masked genome outputted by RepeatMasker. To avoid masking certain repetitive protein families present in the genome, we performed a BLAST (Altschul et al., 1990) search of the *redmask*-produced library against Uniprot. Those repeats with significant hits (evalue <10^-6^) against proteins were removed from the final repeat annotation. Bedtools v2.31.1 (Quinlan and Hall, 2010) was used to produce the final repeat-masked version of the genome.

Gene annotation

Gene annotation was done by combining transcript alignments, protein alignments and *ab initio* gene predictions following the CNAG structural genome annotation pipeline (Figure S1; https://github.com/cnag-aat/Annotation_AAT). Firstly, RNA-Seq reads obtained from several tissues, either sequenced specifically in this study (gill, mantle, foot, haemocytes, and digestive gland) or existing in public databases, were aligned to the genome with STAR v-2.7.10a (Dobin et al., 2013). Transcript models were subsequently generated using Stringtie v2.2.1 (Pertea et al., 2015) on each BAM file and then all the models produced were combined using TACO v0.7.3 (Niknafs et al., 2017). High-quality junctions to be used during the annotation process were obtained by running ESPRESSO (Gao et al., 2023) v1.3.0 after mapping with Minimap2. Finally, PASA assemblies were produced with PASA (Haas et al., 2008) v2.5.2. The TransDecoder program, which is part of the PASA package, was run on the PASA assemblies to detect coding regions in the transcripts. Additionally, the complete proteomes of *C. virginica*, *C. gigas*, *Mytilus coruscus*, *Mytilus galloprovincialis*, and *Mytilus edulis* were downloaded from Uniprot in April 2024 and aligned to the genome using Miniprot v0.6 (Li, 2023). *Ab initio* gene predictions were performed on the repeat-masked *R*. *decussatus* assembly with three different programs: GeneID v1.4 (Parra et al., 2000), Augustus v3.5.0 (Stanke et al., 2006) and Genemark-ES v7.71 (Lomsadze et al., 2014) with and without incorporating evidence from the RNA-Seq data. Geneid and Augustus were specifically trained for this species with a set of 1000 gene candidates obtained from the longest Transdecoder complete models that had a significant BLAST hit against Swissprot (e-value <10-6). Genemark runs in a self-training mode and was not specifically trained with this set of gene candidates. Finally, all the data were combined into consensus CDS models using EvidenceModeler-2.1 (EVM, Haas et al., 2008). Functional annotation was performed on the annotated proteins with Blast2go (Conesa et al., 2005). First, a Diamond blastp (Buchfink et al., 2021) search was made against the nr (last accessed May 2021) and Uniprot (last accessed August 2021) databases. Then, InterProScan (Jones et al., 2014) was run to detect protein domains on the annotated proteins. All these data were combined by Blast2go, which produced the final functional annotation results. Additionally, UTRs and alternative splicing forms were annotated via two rounds of PASA annotation updates. To functionally annotate the proteins of the annotation, the Pannzer’s12 online server was run (Törönen and Holm, 2022).

The annotation of ncRNAs was obtained by running the following steps on the repeat-masked version of the genome assembly. First, the program cmsearch (Cui et al., 2016) v1.1 that is part of the Infernal package (Nawrocki and Eddy, 2013) was run against the RFAM database of RNA families v12.0. Additionally, tRNAscan-SE (Chan et al., 2019) v2.11 was run in order to detect the transfer RNA genes present in the genome assembly. Identification of lncRNAs was done by first filtering the set of PASA-assemblies that had not been included in the annotation of protein-coding genes to retain those longer than 200bp and not covered more than 80% by a small ncRNA. The resulting transcripts were clustered into genes using shared splice sites or significant sequence overlap as criteria for designation as the same gene.

The non-coding RNA annotation required several steps. First, those expressed transcripts that had been assembled by PASA but that had not been annotated as Protein-Coding genes were tagged as long-non-coding RNAs. The reason for this step is that it helps to have putative lncRNAs annotated before using annotation for downstream analysis. However, due to the poor lncRNAs conservation between species, no function was assigned to these lncRNA genes. Moreover, to remove false positives, transcripts overlapping with other Protein-coding genes or repeats were not included into the lncRNA annotation. Finally, only transcripts longer than 200 bp were considered lncRNAs.

The final non-coding annotation contains the lncRNAs and the sncRNAs. The resulting transcripts were clustered into genes using shared splice sites or substantial sequence overlap as criteria for designation as the same gene.

**References**

Altschul, S. F., W. Gish, W. Miller, E. W. Myers, and D. J. Lipman. 1990. “Basic Local Alignment Search Tool.” Journal of Molecular Biology 215: 403–410. <https://doi.org/10.1016/S0022-2836(05)80360-2>.

Buchfink, B., K. Reuter, and H. G. Drost. 2021. “Sensitive Protein Alignments at Tree‐Of‐Life Scale Using DIAMOND.” Nature Methods 18: 366–368. <https://doi.org/10.1038/s41592-021-01101-x>.

gao, X., Z. Lu, S. Wang, J. Jing‐Yan Wang, and X. Gao. 2016. “CMsearch: Simultaneous Exploration of Protein Sequence Space and Structure Space Improves Not Only Protein Homology Detection but Also Protein Structure Prediction.” Bioinformatics 32: i332–i340. <https://doi.org/10.1093/bioinformatics/btw271>.

Gao, Y., F. Wang, R. Wang, et al. 2023. “ESPRESSO: Robust Discovery and Quantification of Transcript Isoforms From Error‐Prone Long‐Read RNA‐Seq Data.” Science Advances 9: eabq5072. <https://doi.org/10.1126/sciadv.abq5072>.

Jones, P., D. Binns, H. Y. Chang, et al. 2014. “InterProScan 5: Genome‐Scale Protein Function Classification.” Bioinformatics 30: 1236–1240. <https://doi.org/10.1093/bioinformatics/btu031>.

Lomsadze, A., P. D. Burns, and M. Borodovsky. 2014. “Integration of Mapped RNA‐Seq Reads Into Automatic Training of Eukaryotic Gene Finding Algorithm.” Nucleic Acids Research 42: e119. <https://doi.org/10.1093/nar/gku557>.

Pertea, M., G. M. Pertea, C. M. Antonescu, T.‐C. Chang, J. T. Mendell, and S. L. Salzberg. 2015. “StringTie Enables Improved Reconstruction of a Transcriptome From RNA‐Seq Reads.” Nature Biotechnology 33: 290–295. <https://doi.org/10.1038/nbt.3122>.

Stanke, M., O. Schöffmann, B. Morgenstern, and S. Waack. 2006. “Gene Prediction in Eukaryotes With a Generalized Hidden Markov Model That Uses Hints From External Sources.” BMC Bioinformatics 7: 62. <https://doi.org/10.1186/1471-2105-7-62>.

Törönen, P., and L. Holm. 2022. “PANNZER —A Practical Tool for Protein Function Prediction.” Protein Science 31: 118–128. <https://doi.org/10.1002/pro.4193>.
